# Antimicrobial peptide activity is anticorrelated with lipid a leaflet affinity

**DOI:** 10.1371/journal.pone.0242907

**Published:** 2020-11-30

**Authors:** Nathaniel Nelson, Belita Opene, Robert K. Ernst, Daniel K. Schwartz

**Affiliations:** 1 Department of Chemical and Biological Engineering, University of Colorado Boulder, Boulder, Colorado, United States of America; 2 Department of Microbial Pathogenesis, School of Dentistry, University of Maryland Baltimore, Baltimore, Maryland, United States of America; Nanyang Technological University, SINGAPORE

## Abstract

The activity of antimicrobial peptides (AMPs) has significant bacterial species bias, the mechanisms of which are not fully understood. We employed single-molecule tracking to measure the affinity of three different AMPs to hybrid supported bilayers composed of lipid A extracted from four different Gram negative bacteria and observed a strong empirical anticorrelation between the affinity of a particular AMP to a given lipid A layer and the activity of that AMP towards the bacterium from which that lipid A was extracted. This suggested that the species bias of AMP activity is directly related to AMP interactions with bacterial outer membranes, despite the fact that the mechanism of antimicrobial activity occurs at the inner membrane. The trend also suggested that the interactions between AMPs and the outer membrane lipid A (even in the absence of other components, such as lipopolysaccharides) capture effects that are relevant to the minimum inhibitory concentration.

## Introduction

The activity of antimicrobial peptides (AMPs) towards different Gram-negative bacteria, while not truly specific, is highly variable in ways that are not easily predictable. The variability of AMPs appears counter-intuitive at first glance, since the mechanism of AMP activity is associated with the degradation, through pores [1] or carpet assembly [2] of the *inner* bacterial membrane, which is composed of a highly conserved phospholipid bilayer. This suggests that the species-bias of AMPs may instead be traced to the outer bacterial membrane [3], which is the first barrier encountered by AMPs, and must be translocated to reach the inner membrane. The outer membrane is asymmetric, with an outer leaflet comprising primarily lipid A (and lipid-A-containing lipopolysaccharides) and a phospholipid inner leaflet [4]. Lipid A exhibits enormous structural variability, in fact lipid A structure and composition can serve as a signature of bacterial identity [5].

The mechanism of AMP activity towards bacteria is complex, comprising binding, translocation, self-assembly, and other processes. It is well established that strong binding to bacterial surfaces is necessary for AMP activity. Measurements of AMP binding to whole bacteria have shown that AMPs show affinity towards bacterial strains upon which they exhibit antimicrobial activity, and also that activity is associated with high AMP surface coverage [6,7]. Biophysical methods, such as NMR and isothermal titration calorimetry have demonstrated that AMPs bind strongly to outer membrane components (e.g. in the form of lipopolysaccharide micelles), and that this binding influences AMP secondary structure [8–11]. Here, we employ a model system to isolate and explore one part of this complex process, the strength of interactions between individual AMP molecules and lipid A. The interest in this particular process was motivated by the fact that biosynthetic modifications of lipid A are often observed to be associated with AMP resistance [12]. Thus, it is plausible to hypothesize that distinctive physico-chemical AMP/lipid A interactions may be related the variable activity of particular AMPs towards different bacteria.

The structure of lipid A comprises 4–7 aliphatic tails (of varying length and saturation), a disaccharide head group, and 0–2 negatively charged phosphate groups [13,14], leading to an enormous number of possible variations. Since most AMPs are positively charged, electrostatic attraction is apparently an important component of AMP/lipid A interactions [15,16]. Hydrophobic interactions have also been shown to influence interactions between AMPs and lipid A. Considering that a given outer bacterial membrane may contain a heterogeneous mixture of lipid A species as well as other lipopolysaccharide components, AMP-lipid A affinity is difficult to predict. Moreover, using traditional methods, it has been challenging to measure isolated AMP interactions with lipid A leaflets in a bilayer geometry. We employed a single-molecule fluorescence imaging method that was capable of quantitatively measuring the affinity between AMPs and hybrid lipid bilayers with a lipid A outer leaflet under steady state conditions, thereby isolating the effects of AMP-lipid A interactions in a relevant geometry.

We previously developed an approach to characterize relevant AMP–lipid A interactions using single-molecule super-resolution imaging to track fluorescently labeled AMPs as they interacted with asymmetric supported lipid bilayer (SLBs) outer membrane mimics [17]. By measuring AMP coverage, adsorption/desorption dynamics, and interfacial diffusion, we showed that this approach was sensitive to subtly different interactions between AMPs and lipid A asymmetric bilayers composed of diphosphoryl and hexa-acylated, monophosphoryl *E*. *coli* lipid A. Here, we employed this approach to characterize the interactions between three different AMPs, and asymmetric bilayers comprising complex lipid A mixtures isolated from four different Gram-negative bacteria: *Acinetobacter baumannii* (AB), *Klebsiella pnemoniae* (KP), *Pseudomonas aeruginosa* (PA), and *Escherichia coli* (EC), and found a distinctive empirical relationship between the strength of these physical interactions and to the activity of AMPs towards the bacterial species from which the lipid A were extracted.

## Materials and methods

The AMPs used in this work–LL37 (LL), Cecropin B (CEC), and Melittin (MEL)–exhibit an amphipathic α-helical structure when interacting with a lipid bilayer but are unstructured in solution [18,19] and have nominal charges of +5, +7, and +6, respectively under physiological conditions. LL37 is a human peptide sequence, Cecropin B is isolated from a giant silk moth, and Melittin is found in bee venom. All three have antimicrobial activity against Gram-negative bacteria; CEC and MEL also show activity against other microbes and tumor cells. We characterized the interactions of these AMPs with asymmetric SLBs incorporating reconstituted lipid A extracted from all four bacterial backgrounds. Structural information about the AMPs is summarized in Table 1.

**Table 1:** Structures of antimicrobial peptides.

Peptide	Primary Sequence	Secondary Structure
LL-37	LLGDFFRKSKEKIGKEFKRIVQRIKDFLRNLVPRTES	primarily helical [20]
Cecropin B	KWKVFKKIEKMGRNIRNGIVKAGPAIAVLGEAKAL	helix-hinge-helix [21,22]
Melittin	GIGAVLKVLTTGLPALISWIKRKRQQ	primarily helical [23]

As described previously [17], we employed a Langmuir-Blodgett/Langmuir-Schaefer (LB/LS) layer-by-layer deposition approach to create asymmetric SLBs with an exposed outer leaflet comprising lipid A and a 1,2-dioleoyl-sn-glycero-3-phosphoethanolamine (DOPE) inner leaflet. In the present study, the lipid A in a given experiment was reconstituted from material isolated from the organism of interest [24]. Large-scale bacterial LPS preparations were isolated using a hot phenol/water extraction method after growth in lysogenic broth supplemented with 1mM MgCl_2_ at 37 ˚C [25]. Subsequently, LPS was treated with RNase A, DNase I and proteinase K to ensure purity from contaminating nucleic acids and proteins [26]. Individual LPS samples were additionally extracted to remove contaminating phospholipids [27] and TLR2 contaminating proteins [28]. Finally, individual LPS preparations were resuspended in 500 ml of water, frozen on dry ice and lyophilized. Lipid A was isolated after hydrolysis in 1% SDS at pH 4.5 as described previously [29].

Lipid bilayers were formed using a LB/LS deposition technique using a NIMA Langmuir trough (KSV NIMA, Espoo, Finland). Lipid A samples were dissolved at a concentration of 1 mg/mL in a 73:24:3 (v:v) mixture of chloroform:methanol:water. DOPE (Avanti Polar Lipids, Alabaster, AL) was dissolved in chloroform at a concentration of 1 mg/mL. Lipids were deposited to the air/water interface by adding 20 mL of lipid A solution or 30 mL of DOPE solution in 2 mL increments. The lipids were then compressed to a surface pressure of 27 mN/m and maintained at 21 ± 0.5 ^o^C. Pieces of 25x25 mm #1 cover glass (ThermoFisher Scientific) were cleaned with hot piranha solution (30% hydrogen peroxide/70% sulfuric acid by volume) at 50°C for 1 h, followed by thorough rinsing in Milli-Q water (Millipore) and drying under ultrapure nitrogen. The dry coverslips were then placed in an ultraviolet (UV)-ozone chamber (PSD series Digital UV Ozone System; Novascan, Ames, IA) for 1 h and used within 20 min of removal from the UV-ozone chamber.

To deposit the inner SLB leaflet, a clean coverslip was first submerged into the Langmuir trough; DOPE was then deposited at the air/water interface. The coverslip, oriented vertical to the interface, was drawn through the lipid monolayer at the air-water interface at a rate of 3 mm/min, depositing a phospholipid monolayer with the head group toward the glass substrate. During monolayer deposition, the surface pressure was held at a constant 27 mN/m through a feedback loop, which yielded monolayers with estimated areas per molecule of 0.55 nm^2^ for DOPE and 1.2 nm^2^ for lipid A. Previous work has shown these to be representative molecular areas for lipids in biological membranes, and appropriate models to study interactions between AMP and lipid A at the air-water interface [30,31]. After inner leaflet deposition, the remaining lipid monolayer at the air-water interface of the Langmuir trough was removed through pipette aspiration. Outer leaflet lipid A was then deposited and compressed using the Langmuir trough. The substrate with deposited inner leaflet was then rotated parallel to the plane of the air/water interface and lowered onto the freshly formed monolayer forming an SLB and passing into the aqueous phase. The resulting SLB was kept submerged in 1x PBS to avoid bilayer disruption.

Peptides were synthesized by GenScript (Piscataway, NJ) with an unnatural azido-lysine amino acid at the C-terminus, enabling fluorescent labeling via copper-free strained alkyne click reaction chemistry [32]. Peptides were dissolved in 1x PBS at room temperature at a concentration of 2 mg/mL. Alexa Fluor 488 dibenzocyclooctyne alkyne (ThermoFisher Scientific, Waltham, MA) was dissolved in dimethyl sulfoxide at a concentration of 2.5 mg/mL. The peptide and fluorophore solutions were mixed at a 3:2 molar ratio of peptide to fluorophore and reacted for 24 h at 4°C. The resultant solution was then passed through a reversed-phase chromatography column (SEC 70 10 x 300 mm column; Bio-Rad, Hercules, CA) at 1 mL/min using PBS and the fluorescent fraction was isolated.

Super-resolution single-molecule total internal reflection fluorescence microscopy was employed to localize and track individual fluorescently labeled AMP molecules as they adsorbed to, diffused on, and desorbed from the bilayer surface. Time series of images, with 100ms acquisition time, were captured on an inverted Nikon Eclipse Ti Microscope with a Plan-Apo 100x 1.45 NA oil immersion objective (Nikon, Melville, NY) using a Hamamatsu CMOS camera. Excitation light generated by a 100 mW 491 nm solid-state laser (Cobolt Calypso 100; Cobolt) was passed through a total internal reflection illuminator (TI-TIRF-EM; Nikon) to generate an evanescent field at the SLB-water interface with incident energy of ~0.75 kW/cm^2^. All microscopy experiments were performed at 21 ± 0.5 ^o^C. At least three replicate experiments were performed for each experimental condition. Each experiment comprised at least five movies, with durations of approximately 10s, from independent fields of view. Single-molecule tracking was performed using a custom *Mathematica* script to identify and localize diffraction-limited spots [33,34]. At least 2,000 molecular trajectories were obtained for each experimental condition. The number of molecules was determined in each frame and used to calculate the average surface coverage. The standard error from replicate experiments was used to determine the uncertainty in the average surface coverage.

We focused on observations of the steady-state (equilibrium) surface density of AMPs (i.e., the adsorbed AMP mass per unit bilayer area) as it related to the concentration of AMP in solution. To measure the affinity of AMPs to lipid A leaflets at the low-concentration/low-surface density limit, AMPs were added to PBS (pH 7.4) buffer in contact with SLBs at a concentration of 10^−10^ M (which allowed for single-molecule localization) and allowed to equilibrate. Image sequences were then acquired in multiple locations, where the duration of the movies was short compared to the characteristic time constant for bleaching as in previous work. Adsorbed AMPs were localized and counted to determine the average equilibrium number of molecules adsorbed per surface area and converted to mass per area using the AMP molecular mass. The affinity was then calculated as the ratio of the AMP surface density to solution concentration:

$$\mathrm{Affinity}=\frac{\mathrm{AMP}\;\mathrm{surface}\;\mathrm{density}\;(\mathrm{mg}/m^{2})}{\mathrm{solution}\;\mathrm{concentration}\;(\mathrm{mM})}$$

Given the extremely low surface density (individual AMP molecules were typically separated by μm distances), and the fact that no significant differences in fluorescence intensity were observed between localized objects, AMP were presumed to be monomeric, which is an advantage of this SM approach in measuring surface affinity.

## Results and discussion

**Fig 1:**
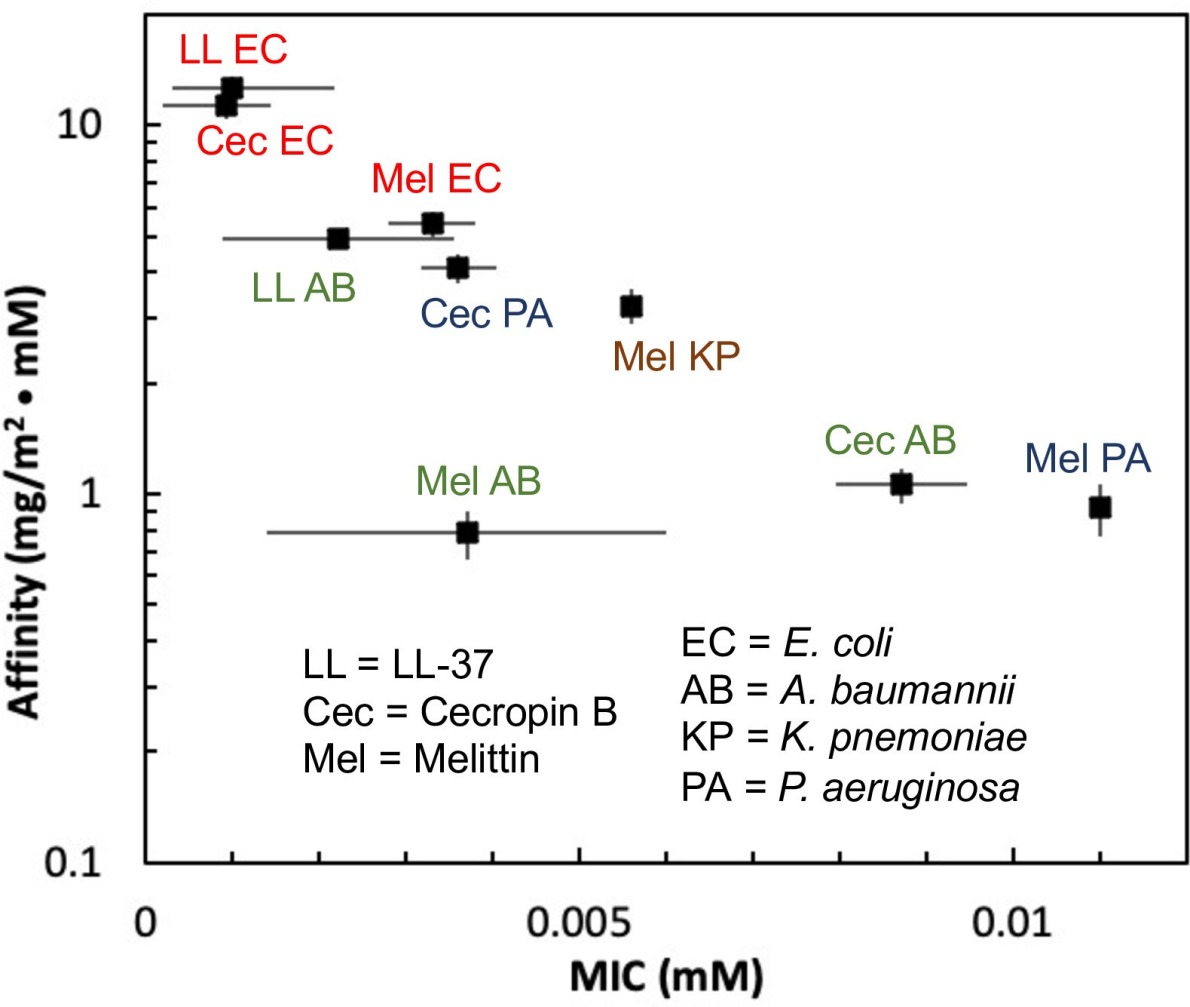
Semi-logarithmic plot of AMP affinity from single-molecule observations of fluorescent AMPs adsorbed on asymmetric hybrid bilayers composed of lipid a isolated from gram-negative bacteria. The data are plotted vs. the average of previously published MIC values. Table 2 summarizes the sources of the MIC values. The horizonal bars represent the full range of published values. The vertical bars represent the uncertainty in the mean affinity from replicate experiments as described in the main text. The numerical values of these data are tabulated in S1 Table in the Supporting Information.

Fig 1 illustrates the main result of this article, plotting the measured AMP affinity vs. minimum inhibitory concentrations (MIC) associated with the antimicrobial activity of the same AMPs against the organisms from which the lipid A was extracted, for nine different AMP/lipid A combinations. The MIC values were drawn from a literature review, summarized in Table 1, and reflect the variability of published MIC measurements. Each average MIC is indicated by a symbol, and the range of MIC values is spanned by the connected bars. With the exception of Melittin interaction with lipid A isolated from *A*. *baumannii*, the affinity exhibits a systematic anti-correlation with measured MIC values over more than one order of magnitude in both quantities. This empirical trend is consistent with the conjecture that the species bias of AMP activity is related to AMP interactions with bacterial outer membranes, despite the fact that the mechanism of antimicrobial activity occurs at the inner membrane. Moreover, while the outer membrane exhibits a complex structure, the trend also suggests that the interactions between AMPs and the outer membrane lipid A (even in the absence of other components, such as the carbohydrate portion of lipopolysaccharides) captures interactions that are relevant to the minimum inhibitory concentration. This behavior is expected to be related to differences in lipid A structure among species and to complex interactions that are related to charge, hydrophobicity, membrane heterogeneity, and other factors.

**Table 2:** Sources of minimum inhibitory concentration values used in Fig 1.

Bacterium	Peptide	Sources of MIC values
*Acinetobacter baumannii*	Melittin	4 mg/L [35], 17 mg/L [36]
	LL-37	16 mg/L [37], 4 mg/L [38]
	Cecropin B	32 mg/L [35], 7.04 μM [39]
*Escherichia coli*	Melittin	8 mg/L [40], 3.8 μM [41]
	LL-37	2.1 mg/L [42], 9.8 mg/L [43], 0.312 μM [44]
	Cecropin B	0.2 μM [45], 1.17 μm [46], 1.43 μM [39]
*Pseudomonas aeruginosa*	Melittin	32 mg/L [40]
	Cecropin B	4 μM [45] 3.13 μM [46]
*Klebsiella pnemoniae*	Melittin	16 mg/L [40]

It is possible that the fluorescent probe may have a modest influence on the interactions between the labeled peptide and lipid A; indeed this is a fundamental limitation of all fluorescence methods. However, since the same fluorescent label and conjugation method has been used in all cases, and the molecular weight of the label is small compared to that of the AMPs, it is plausible to infer that while the absolute affinities may be slightly perturbed due to the labeling, the general trends of affinity should still be representative of those associated with unlabeled AMPs.

SM-tracking experiments were also performed at higher solution AMP concentrations, similar to the MIC for each AMP-lipid A combination. In these experiments, the fluorescently labeled AMP concentration was maintained at the same low level to enable SM localization, and large amounts of unlabeled AMPs was added to bring the total concentration to the relevant values. The total surface density was then calculated by multiplying the apparent surface density of labeled AMP by the ratio of total AMP to labeled AMP. Even at higher concentrations corresponding to the MIC, the total surface coverage was found to be in the low coverage regime, where surface coverage is proportional to solution concentration. The values of surface coverage were in the range 0.003–0.018 mg/m^2^, which represents less than 1% of a close-packed peptide monolayer.

In the context of classical Langmuir adsorption theory, the affinity is related to the equilibrium constant, *K∝exp*(*U_ads_/k_B_T*), where *U*_*ads*_ is the potential energy of adsorption, *k*_*B*_ is Boltzmann’s constant, and *T* is the absolute temperature. Thus, roughly speaking, ln*K* may be expected to provide information about the adsorption energy. The semi-logarithmic form of Fig 1 leads one to speculate that ln *K*∝MIC, which would be the case if the MIC were proportional to the adsorption energy of the AMP on the lipid A leaflet. While there is currently no existing theoretical basis to understand the connection of AMP adsorption to bacterial activity, this empirical relationship may form the basis of a plausible hypothesis for future testing and to guide theory.

## Conclusions

The results shown Fig 1 demonstrate an empirical relationship between a *physical* measure of intermolecular interactions and a quantity representing *biological* activity. In particular, they show that the interfacial affinity of a given AMP to a lipid bilayer with a lipid A outer leaflet is anticorrelated with the activity of that AMP towards the Gram negative bacterial species from which the lipid A was extracted. That is, AMPs that interact more strongly with the lipid A in the bacterial outer membrane require a smaller dose to impart an inhibitory effect, and vice versa.

These data, while shown concisely in a single figure, represent a comprehensive study incorporating experiments that include three different AMPs and lipid A from four different bacterial organisms. Moreover, the trend connecting the values of affinity and MIC spans a substantial range of more than one order of magnitude in both quantities. This previously unknown relationship provides support for the hypothesis that AMP species bias is directly related to outer membrane interactions, with lipid A in particular, despite the fact that the effects of AMPs are known to occur at the inner membrane.

Presumably, the affinity measured here can be physically related to molecular level interactions between AMP and species-specific lipid A molecules. These interactions may be complex, and not simply related to one particular type of force. For example, the affinity between an AMP and a lipid A layer may involve a combination of electrostatic, hydrophobic, and other non-covalent effects, which can potentially be probed using computational methods such as atomistic molecular dynamics simulations. The results presented here may stimulate computational studies that can isolate these interactions.

Importantly, the interactions between AMP molecules and lipid A leaflets reported here clearly represent only one small piece of a complex and diverse process that involves many steps and multiple molecular components. Our hope is that the empirical relationship identified in this manuscript will complement other biophysical studies of AMPs that employ various methods to explore interactions with other cellular components and structures, ultimately leading to a more comprehensive understanding.

## Supporting information

S1 Table Tabulated data displayed in Fig 1 of the text. (DOCX)
