# Supplementary material for: Antimicrobial peptide activity is anticorrelated with lipid a leaflet affinity
Source: PLoS One. 2020 Nov 30;15(11):e0242907. doi: 10.1371/journal.pone.0242907 (PMC7703904; doi:10.1371/journal.pone.0242907)
Supplement: S1 Table — (DOCX) [file pone.0242907.s001.docx]

**Antimicrobial Peptide Activity is Anticorrelated with Lipid A Leaflet Affinity**

Nathaniel Nelson^1^, Belita O’Pene^2^, Robert K. Ernst^2^, and Daniel K. Schwartz^1,^*

^1^ Department of Chemical and Biological Engineering, University of Colorado Boulder, Boulder, Colorado

^2^ Department of Microbial Pathogenesis, School of Dentistry, University of Maryland Baltimore, Baltimore, Maryland

*Corresponding author: [daniel.schwartz@colorado.edu](mailto:daniel.schwartz@colorado.edu)

**SUPPORTING INFORMATION**

**S1 Table: Tabulated data displayed in Figure 1 of the text.**

| **AMP** | **Bacterium** | **Average MIC (mM)** | **MIC range (mM)** | **Affinity (mg m^-2^ mM^-1^)** |
| --- | --- | --- | --- | --- |
| LL-37 | *E. coli* | 0.000987 | 0.00031–0.0022 | 12.6 ± 0.9 |
| Cecropin B | *E. coli* | 0.000933 | 0.00020–0.0014 | 11.3 ± 0.8 |
| Melittin | *E. coli* | 0.0033 | 0.0028–0.0038 | 5.4 ± 0.4 |
| Melittin | *A. baumannii* | 0.0037 | 0.0014–0.0060 | 0.78 ± 0.12 |
| Cecropin B | *P. aeruginosa* | 0.0036 | 0.0032–0.0040 | 4.1 ± 0.4 |
| LL-37 | *A. baumannii* | 0.0022257 | 0.00089–0.0036 | 4.9 ± 0.4 |
| Melittin | *K. pnemoniae* | 0.0056 | 0.0056 | 3.2 ± 0.4 |
| Cecropin B | *A. baumannii* | 0.0087 | 0.0079–0.0095 | 1.05 ± 0.12 |
| Melittin | *P. aeruginosa* | 0.011 | 0.011 | 0.91 ± 0.15 |

**References**

1. Vila-Farres, X.; de la Maria, C. G.; Lopez-Rojas, R.; Pachon, J.; Giralt, E.; Vila, J., In vitro activity of several antimicrobial peptides against colistin-susceptible and colistin-resistant Acinetobacter baumannii. *Clin Microbiol Infec* **2012,** *18* (4), 383-387.

2. Rangel, K.; Lechuga, G. C.; Souza, A. L. A.; da Silva Carvalho, J. P. P.; Villas Bôas, M. H. S.; De Simone, S. G., Pan-Drug Resistant Acinetobacter baumannii, but Not Other Strains, Are Resistant to the Bee Venom Peptide Melittin. *Antibiotics* **2020,** *9*, 178.

3. Feng, X. R.; Sambanthamoorthy, K.; Palys, T.; Paranavitana, C., The human antimicrobial peptide LL-37 and its fragments possess both antimicrobial and antibiofilm activities against multidrug-resistant Acinetobacter baumannii. *Peptides* **2013,** *49*, 131-137.

4. Jaskiewicz, M.; Neubauer, D.; Kazor, K.; Bartoszewska, S.; Kamysz, W., Antimicrobial Activity of Selected Antimicrobial Peptides Against Planktonic Culture and Biofilm of Acinetobacter baumannii. *Probiotics Antimicro* **2019,** *11* (1), 317-324.

5. Xia, L. J.; Liu, Z. Y.; Ma, J.; Sun, S. R.; Yang, J. H.; Zhang, F. C., Expression, purification and characterization of cecropin antibacterial peptide from Bombyx mori in Saccharomyces cerevisiae. *Protein Expres Purif* **2013,** *90* (1), 47-54.

6. Dosler, S.; Karaaslan, E.; Gerceker, A. A., Antibacterial and anti-biofilm activities of melittin and colistin, alone and in combination with antibiotics against Gram-negative bacteria. *J Chemotherapy* **2016,** *28* (2), 95-103.

7. Pandey, B. K.; Ahmad, A.; Asthana, N.; Azmi, S.; Srivastava, R. M.; Srivastava, S.; Verma, R.; Vishwakarma, A. L.; Ghosh, J. K., Cell-Selective Lysis by Novel Analogues of Melittin against Human Red Blood Cells and Escherichia coli. *Biochemistry-Us* **2010,** *49* (36), 7920-7929.

8. Turner, J.; Cho, Y.; Dinh, N. N.; Waring, A. J.; Lehrer, R. I., Activities of LL-37, a cathelin-associated antimicrobial peptide of human neutrophils. *Antimicrob Agents Ch* **1998,** *42* (9), 2206-2214.

9. Luo, Y.; McLean, D. T. F.; Linden, G. J.; McAuley, D. F.; McMullan, R.; Lundy, F. T., The Naturally Occurring Host Defense Peptide, LL-37, and Its Truncated Mimetics KE-18 and KR-12 Have Selected Biocidal and Antibiofilm Activities Against Candida albicans, Staphylococcus aureus, and Escherichia coli In vitro. *Front Microbiol* **2017,** *8*.

10. Gunasekera, S.; Muhammad, T.; Stromstedt, A. A.; Rosengren, K. J.; Goransson, U., Backbone Cyclization and Dimerization of LL-37-Derived Peptides Enhance Antimicrobial Activity and Proteolytic Stability. *Front Microbiol* **2020,** *11*.

11. Romoli, O.; Mukherjee, S.; Mohid, S. A.; Dutta, A.; Montali, A.; Franzolin, E.; Brady, D.; Zito, F.; Bergantino, E.; Rampazzo, C.; Tettamanti, G.; Bhunia, A.; Sandrelli, F., Enhanced Silkworm Cecropin B Antimicrobial Activity against Pseudomonas aeruginosa from Single Amino Acid Variation. *Acs Infect Dis* **2019,** *5* (7), 1200-1213.

12. Wang, J. R.; Ma, K.; Ruan, M. S.; Wang, Y. J.; Li, Y.; Fu, Y. V.; Song, Y. H.; Sun, H. B.; Wang, J. F., A novel cecropin B-derived peptide with antibacterial and potential anti-inflammatory properties. *Peerj* **2018,** *6*.
